# Healthcare Access and Outcomes for Refugee Women in Transit: A Scoping Review of Facilitators and Barriers in South and Southeast Asia

**DOI:** 10.1007/s10903-025-01722-w

**Published:** 2025-06-30

**Authors:** Gabriela Fernando, Asiyah Nida Khafiyya, Anak Agung Istri Diah Tricesaria, Jessica Watterson, Sabina Satriyani Puspita

**Affiliations:** 1 Public Health, Monash University, Banten, Indonesia; 2 Public Health Department of Community Medicine, Faculty of Medicine, Universitas Islam Negeri Syarif Hidayatullah Jakarta, Banten, Indonesia; 3 Human Geography, Anthropology and Development Studies Department, School of Social Sciences, Faculty of Arts, Monash University, Melbourne, Australia; 4 School of Health and Social Development, Deakin University, Melbourne, Australia; 5 Jeffrey Cheah School of Medicine and Health Sciences, Monash University, Kuala Lumpur, Malaysia; 6 Public Policy and Management, Monash University, Banten, Indonesia

**Keywords:** Transit refugees, Women’s health, Healthcare access, Health equity, Migration

## Abstract

Forced displacement has reached unprecedented levels globally, with women and girls comprising over half of the refugee population. In South and Southeast Asia, transit refugee women face unique health challenges, yet their health experiences and needs remain underexplored and underserved. This review explores the existing evidence on the health experiences and healthcare access of transit refugee women in these regions. A search was conducted using PubMed [MEDLINE], Scopus, Google Scholar, alongside UNHCR, WHO, and IOM databases between October 2023 and January 2024. Peer-reviewed and grey literature published from 2013 to 2024 that analysed health outcomes and experiences of transit refugee women in South and Southeast Asia were included. A narrative synthesis was used to identify the major themes related to the health outcomes and healthcare access of transit refugee women. Fourteen articles met the inclusion criteria, of which a majority of the studies were from Bangladesh, Thailand-Myanmar border, and Malaysia, mainly drawing insights from Rohingya, Karen, and Chin communities. Key health issues included sexual and reproductive health, including family planning, contraceptive use, HIV transmission, sexual and gender-based violence, and mental health. Distance and availability of health services, spousal consent for health-seeking, out-of-pocket health expenditure, cultural and religious beliefs, and stigmatization were barriers to accessing healthcare. These barriers may contribute to foregoing contraceptive usage, poor family planning, poor HIV-related health knowledge transmission, high psychological distress, and poor mental health outcomes. However, targeted interventions such as having the UNHCR card, the availability of health clinics within camps, recruiting female health workers, provision of counselling and health education programs, can positively influence health outcomes, healthcare seeking and utilization behaviours, and health knowledge transmission. Transit refugee women in South and Southeast Asia face significant challenges due to unmet health needs and limited healthcare access. These insights highlight the need for future health research, programs and policy action to better integrate targeted, gender-responsive interventions that can enhance health access of this vulnerable subgroup regionally.

## Background

Globally, one in eight individuals is currently a migrant or forcibly displaced [1]. By the end of September 2023, a record 114 million people were displaced by war, persecution, human rights violations, and environmental crises [2]. South and Southeast Asia serve as critical transiting hubs connecting countries of origin to final destinations for millions of refugees fleeing from such atrocities. Over 280,000 refugees are residing across Malaysia, Thailand, and Indonesia and over 1.3 million reside in Bangladesh and India [3–5]. Despite the significant role of these regions in global migration pathways, there remains a dearth of research and policy focusing on the health and healthcare access of transit refugees, particularly for women who face disproportionate exposure to health and social risks [6, 7].

### Women Refugees in Transit: A Vulnerable and Overlooked Subgroup

While there is no universal definition categorizing this subgroup, transit refugees are individuals who, having fled their country of origin, are temporarily displaced while awaiting resettlement or asylum in a final host country. This subgroup differs from asylum seekers, who are awaiting a decision on their asylum application, and resettled refugees, who have been granted protection in a third country [8]. It also includes individuals whose legal documentation has expired, leaving them in a prolonged state of uncertainty and vulnerability, often with limited access to formal protection, legal rights and services. Despite the implication of short-term retention suggested by the terms ‘transit’ and ‘transit routes’, studies reveal that this period can extend from several months to several years [9–11].

Studies have highlighted that refugees face distinct vulnerabilities at each stage of migration—pre-departure, during the journey, in transit, and in host countries—with the transit context significantly heightening risks of exploitation, kidnappings, torture and sexual violence, and severe mental and physical trauma and health inequities, particularly for women [10, 12]. These risks are further compounded by gendered barriers and systemic neglect within transit contexts, including a lack of- or poor living conditions, with some living within detention camps and others in blended community contexts, often with little access to legal recourse or health and psychological support.

Sexual and reproductive health and rights (SRHR) are often neglected due to inadequate health systems’ responses in transit regions, alongside cultural or legal constraints, with women frequently encountering barriers to accessing contraception, with complications from cultural practices such as female genital mutilation (FGM) and consequential obstetric health complications, poor maternal health services, unsafe abortions, and increased incidences of sexually transmitted infectious (STIs) [7, 13, 14]. Moreover, studies have reported that between men and women, transit conditions exacerbate the risk of sexual and gender-based violence (SGBV) for women, with every one in five women refugees experiencing forms of violence, including sexual assault, exploitation, and intimate partner violence [15–17]. Factors such as age of migration, marital status, and days of preparation for the travel (a measure of suddenness of departure) are found to be associated with the risk of exposure and vulnerability of girls and women, particularly in relation to SGBV [17, 18]. Consequently, these vulnerabilities have contributed to surging rates of child marriage and teenage pregnancies within these transit environments [19].

Chronic health conditions, such as diabetes and hypertension, are also poorly managed due to disruptions in care, lack of medications, and prioritization of acute health needs in crisis settings [20, 21]. Mental health challenges are pervasive, with women transit refugees reporting high levels of trauma, depression, and suicidal ideation, often compounded by stigma and the absence of culturally sensitive mental health services [10, 22]. While the relationship between women’s chronic health conditions and mental health challenges has not been explicated, untreated chronic health conditions may factor in women’s mental health challenges and vice versa [23].

### Emergence of Global Evidence Versus Evidence Gaps in South and Southeast Asia

Emerging evidence and policy innovation focus on transit contexts in predominantly Europe and the Mediterranean. Health programming and policies in these regions have begun addressing health inequities and expanding access to healthcare, particularly for women refugees in transit [24]. However, similar efforts in South and Southeast Asia remain sparse, creating a significant gap in addressing the needs of this highly vulnerable population. This gap is further exacerbated by the fact that the majority of South and Southeast Asia countries are not signatories to the *1951 Refugee Convention or its 1967 Protocol*, leaving refugees vulnerable to exploitation, limited access to services, and uncertain legal status. It also places significant strain on national capacities for hosting, financing, and delivering services to meet the needs of refugees, particularly those in transit, as well as host populations.

A study by Quigley et al. [25] that focused on the health system challenges and responses of refugees across Indonesia, Malaysia, Myanmar and Thailand noted that financial, juridico-political and sociocultural barriers, alongside the lack of national commitment to universal health coverage (UHC), contribute to unmet health needs. These include significant mental trauma, untreated chronic conditions, starvation and malnutrition, exposure to infectious diseases such as tuberculosis due to conditions of detention camps, and injury from abuse and sexual violence. The study also highlights the role of NGOs and community-based organizations in providing health services, advocacy, and education to refugee populations. Yet, despite the increasing use of these regions as transit hubs, the specific health needs and healthcare access challenges faced by these transit refugee women remain underexplored.

Therefore, utilizing a narrative synthesis, this review aims to scope the existing evidence to explore the health outcomes, healthcare access and experiences of refugee women in transit across South Asia and Southeast Asia. By highlighting these issues, the review seeks to inform future health interventions and policy action, contributing to more effective support systems for refugees in perilous transit conditions, both regionally and globally.

### Positionality Statement

None of the authors involved in this review were refugees themselves. The authors were faculty in Schools of Public Health, Medicine and Health Sciences, and Public Policy, as well as a graduate student in the School of Social Sciences. Most authors work and engage with private and urban universities in South and Southeast Asia, including in Australia. Most authors identify as women, and have direct experience working with the refugee population in Indonesia. The authors believe that including this positionality statement, despite this article being a literature review, is essential to respecting the agency of refugees and critically reflecting on their own biases.

## Methods

### Search Strategy and Selection Criteria

The search was conducted between October 2023 and January 2024, across multiple databases, including PubMed [MEDLINE], Scopus, Google Scholar, and the e-libraries of the International Organization for Migration (IOM), World Health Organization (WHO), and United Nations High Commissioner for Refugees (UNHCR). The search focused on peer-reviewed articles only in English language that analysed the health outcomes and experiences of adult refugee women (18 + years of age) with transit status residing across South Asia (List of countries by World Bank categorization: Bangladesh, Bhutan, India, Maldives, Nepal, Pakistan, Sri Lanka, and Afghanistan) and Southeast Asia (List of countries by World Bank categorization: Brunei, Cambodia, Indonesia, Laos, Malaysia, Myanmar, the Philippines, Singapore, Thailand, and Vietnam). The articles were also restricted to those published after 2013 to date. Articles published after 2013 are included to capture the evolving discourse around refugee and migrant policies in the lead up to and following the adoption of the *UN General Assembly’s New York Declaration for Refugees and Migrants* in 2016 [26]. This period reflects key shifts in global frameworks and responses to refugee and migration crises.

A list of predetermined key terms using a combination of MeSH and non-MeSH terms that captured ‘transit refugee’, ‘health access’ and various ‘health outcomes’, ranging from infectious diseases, sexual and reproductive health and rights, maternal health, physical injuries, non-communicable diseases, mental health, and gender-based violence, from relevant public health literature and the WHO’s Global Action plan on promoting the health of refugees and migrants [27] (see Appendix). Two reviewers (AK and GF) developed the list of search terms and the eligibility criteria, and Boolean operators were used to combine these terms in the search.

### Data Extraction

In the initial screening phase, titles and abstracts of identified articles were screened based on the predefined eligibility criteria (see Table 1). Articles that met the criteria or showed potential for meeting them were advanced to a full-text screening stage. In this subsequent phase, each article was carefully reviewed to ensure full compliance with the eligibility criteria. Only those that were relevant to the review topic and focused on the specified health outcomes were included in the final review. Key indicators such as publication year, country and region, sample size, study design, and key outcomes were manually extracted from each article. Five independent reviewers conducted the full-text screening, appraised the quality of articles using an adaptation of the Joanna Briggs Institute’s appraisal tool (see Appendix), and performed the data extraction. Any disagreements regarding the eligibility, quality, and the scope of the articles were resolved through an iteration of discussions among the reviewers.

**Table 1:** Eligibility criteria for the review

Criteria items	Articles included	Articles excluded
Type of articles	Peer reviewed literature (i.e., qualitative, quantitative, mixed-methods analyses), peer-reviewed grey literature (e.g., Working papers)	Audio recordings, visuals/graphics, book chapters, review articles (systematic literature reviews, scoping reviews), editorials/commentaries/policy briefs/thesis or dissertation reports, simulations/modelling studies, abstracts only (e.g., conference abstracts)
Definition of transit	According to the International Organization for Migration [8]: Country of transit: In the migration context, the country through which a person or a group of persons pass on any journey to the country of destination or from the country of destination to the country of origin or the country of habitual residence Refugees in transit: Refugees who are temporarily admitted in the territory of a State under the condition that they are resettled in Posselt [28] defined “Transitional contexts” as those in which the participants did not possess legal residency status nor were they residing in host countries which offered a standard UNHCR resettlement programme	
Year of publication	Articles only published between 2013 and 2024	Articles published before 2013
Study population and geographical restriction	Articles on adult refugee/asylum seeker/undocumented migrant women (above 18 + years of age) residing in ‘transit’ conditions Articles only from South Asia and Southeast Asia (list of countries: Bangladesh, Bhutan, India, Maldives, Nepal, Pakistan, Sri Lanka, Afghanistan, Brunei, Cambodia, Indonesia, Laos, Malaysia, Myanmar, the Philippines, Singapore, Thailand, and Vietnam)	Articles on refugees or asylum seekers residing in host countries with legal status Articles not from South Asia or Southeast Asia Articles in which refugees made up less than 25% of the study population Studies on children/youth or women below 18 years of age
Language restriction	Literature available in English	Literature published in other languages
Outcome measures	Health area focus related to the following: infectious diseases or communicable diseases, physical injury or wounds, psychological symptoms and mental health, Non-communicable diseases or chronic diseases, sexual and reproductive health and rights, maternal health and pregnancy, sexual and gender-based violence and violence against women Health behaviours (health seeking, health promoting, healthcare utilization) Healthcare access (quality, access, acceptability, affordability, availability) Health knowledge/awareness	Analyses on disease profile or epidemiology only Analyses on molecular/genetic analyses Analyses on health care providers’ perceptions only

### Data Analysis

This review employed a narrative synthesis approach, which involved the identification and grouping of themes related to health outcomes and healthcare access. In addition to these primary themes, the analysis also highlighted intersectional perspectives, including gender perspectives that may influence the health and healthcare access of transit refugee women.

## Results

**Fig. 1:**
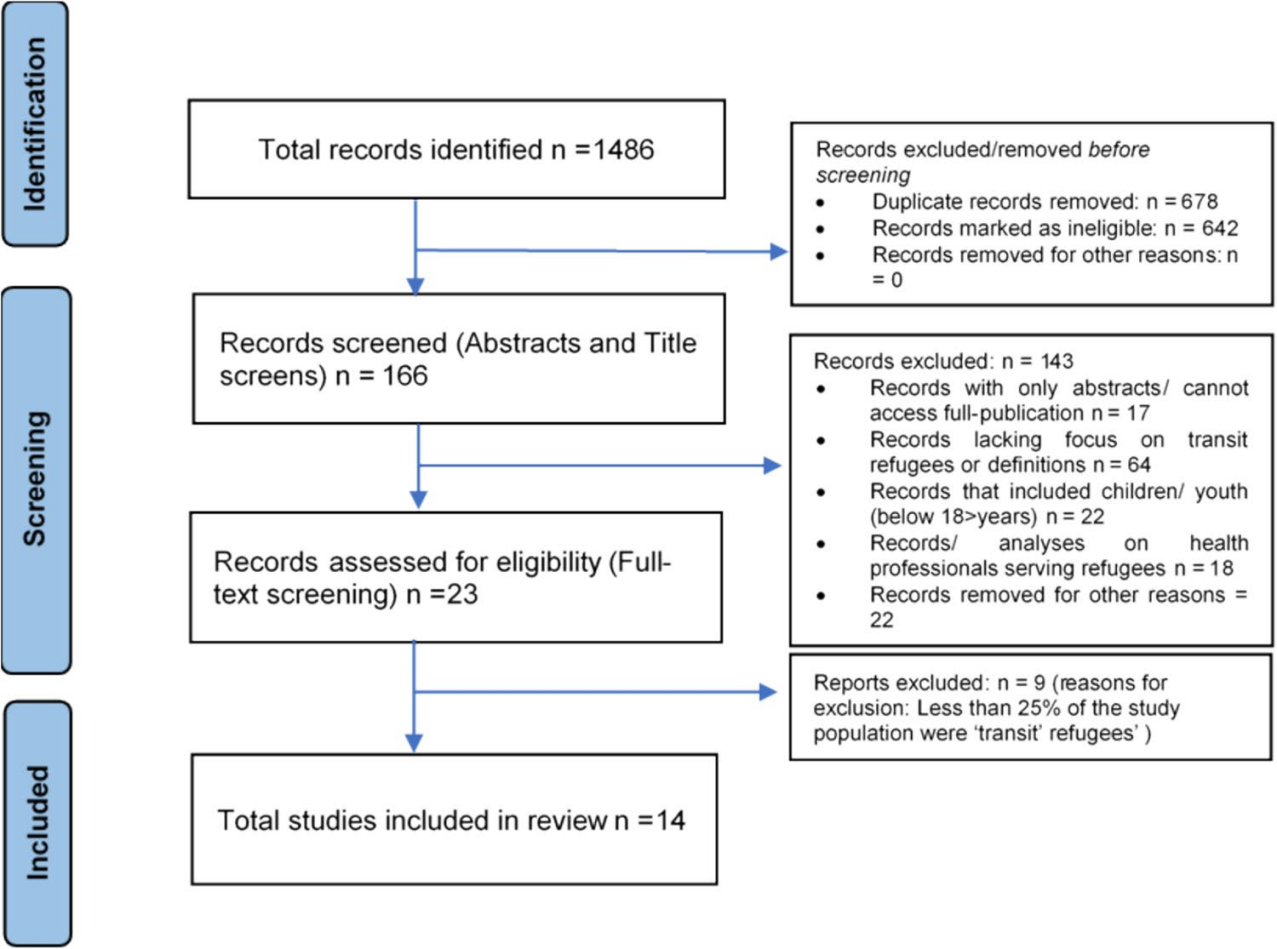
Identification, screening and the inclusion of articles in the review

This review, using a narrative analysis, aimed to scope the existing evidence on the health outcomes, healthcare access, and experiences of refugee women in transit across South Asia and Southeast Asia. From the initial set of 1486 records, a total of 14 articles were identified for inclusion following the full-text screening (see Fig. 1). Table 2 summarises the key findings from the review (see Appendix).

A majority of the articles captured insights from transit refugee women living in Bangladesh [26–30], Thailand [34–41], and Malaysia [42]. Transit refugee women captured in this review originated only from Myanmar, with Rohingya and Karen being the main ethnic groups. All studies from Bangladesh focused exclusively on the Rohingya population temporarily residing in Kutupalong refugee camp, the closest part of Bangladesh to Rakhine State in Myanmar. Meanwhile, most studies from Thailand assessed a mix of ethnic groups such as Chin and Karen communities who fled Southern Myanmar and were living in transit at the Mae La refugee camp in Thailand, along with groups who held different legal statuses such as asylum seekers and migrants. Lastly, the study from Malaysia observed Rohingya refugees in transit who live in urban areas and are often referred to as urban refugees because they live among the local communities instead of a camp.

Of the included articles, two were qualitative studies, eight were quantitative studies, and four utilized mixed-methods approaches. The majority of articles gathered primary data by recruiting participants from the community settings or clinics that serve the refugee camps, whereas two studies conducted retrospective analyses of records from clinics or non-governmental organizations (NGOs) working with refugees in transit [30, 41].

### Health Outcomes

A majority of the articles [27, 29–38] reported on SRHR, including three studies on family planning and contraceptive use [30, 33, 41], one on HIV [29], and two in maternal and neonatal health [32, 40]. Meanwhile, four studies focused on mental health [32–35] and two studies [39, 42] focused on SGBV. Three studies [31, 33, 42] captured a combination of SRHR outcomes, consisting of family planning, STIs, menstrual health and hygiene, maternal health, and SGBV.

In regards to family planning and contraceptive use, Khan et al. [30] reported in a study from Bangladesh that contraceptive injections were the most commonly used type of contraceptive, followed by the oral contraceptive pill, and implants and sterilization were used the least. Additionally, a study from the Thai-Myanmar border [41] reported an increase in contraceptive use between 2002 and 2015, which was associated with new contraceptive consultations. This also resulted in reduced rates of pregnancy during this period.

Fellmeth et al. [35–38] reported that perinatal mental health symptoms included intense sadness, anger and fear, excessive worry, depression, and suicidal ideation, some of which were associated with factors such as employment and income insecurity, lack of family and social support, and having experiences of trauma and violence. Refugees were more likely to experience suicidal ideation, even though they are at a lower risk of experiencing depression, compared to migrants [38]. Additionally, unplanned pregnancy also increased the experiences of suicidal ideation among women [40].

Rajaratnam et al. [42] reported that SGBV manifests in the form of marital rape, domestic violence, child rape and sexual assaults, with reported perpetrators included their spouses, community members, locals, employers and teachers. SGBV was highlighted as a barrier to healthcare utilization, as a factor that heightened the risk of moderate to severe perinatal depression, and increased the odds of pregnancy complications such as vaginal bleeding and severe abdominal pain [39].

### Facilitators and Barriers to Healthcare Knowledge and Utilization

The analysis highlighted several factors that facilitated and barricaded access to healthcare, including the availability of healthcare services, healthcare financing and resources, and health knowledge transmission. These factors positively and negatively influenced preventative health behaviours, health-seeking and healthcare utilization behaviours related to several health outcomes such as antenatal care, HIV transmission, family planning, and contraceptive use.

Six articles [29–34] highlighted that distance to healthcare facilities and the availability of healthcare facilities and services improved health knowledge, health-seeking and healthcare utilization behaviours related to antenatal care, family planning, contraceptive use, and HIV transmission. In Cox’s Bazar, Bangladesh, the availability of healthcare facilities within the residential block increased the odds of having better HIV knowledge by twofold and the use of contraceptives by almost fourfold among transit refugee women [29, 30].

Additionally, having regular interaction with healthcare providers, including community health workers and health volunteers, was noted in five studies as a facilitating factor for healthcare utilization and knowledge [27–31, 36]. Various forms of interaction included consultations regarding SRHR [31–33], door-to-door visits [30, 31], participating in interventions such as family planning, counselling and SRHR awareness programs at the camp [28, 30], regular visits to health clinics [33], including antenatal care (ANC) visits, and prior utilization of family planning services [34]. These facilitating factors resulted in increased utilization of ANC that corresponds with reduced low birthweight prevalence, with one study highlighting that the number of ANC visits served as a protective factor against low birthweight delivery [34].

Regular interaction with health professionals through door-to-door visitations and home visits, and having a reliable source of knowledge were associated with improved health awareness, and contraceptive use and contributed to having better awareness and perception towards SRHR interventions [31]. However, three studies [31–33] highlighted that cultural attitudes and social stigma—such as norms discouraging open discussion of sexual and reproductive health, and beliefs that contraception use is morally inappropriate or contrary to religious values—within communities and by health professionals, were associated with poor sexual and reproductive health outcomes, such as reduced contraceptive usage and family planning utilization.

Religious and cultural beliefs were highlighted as a barrier in five studies [29–33], such as that men and women were prohibited from interacting and sharing the same space in ambulances and hospitals [29]. This impacted women’s menstrual health and hygiene practices, as well as maternal health outcomes, including birthing at health facilities. Rohingya women also believed that using contraceptives was a religious sin, resulting in the disuse of contraceptives and family planning. Additionally, the belief that having more children, particularly male children, brought financial solvency and security as they age, also reduced family planning and contraceptive usage [33]. Additionally, three studies [29, 32, 33] reported that poor health knowledge, health misinformation and misperceptions (e.g., probable side effects of contraceptives) attributed to women’s refusal to use contraceptives.

The employment status of women and mobility outside their shelter were facilitating factors for seeking healthcare [29, 30, 33]. Women who were employed had higher odds of exhibiting good knowledge of HIV and displayed higher use of contraceptives [29, 30]. Other demographic characteristics such as age when first married, having a higher education level, the employment status of their spouse, and the absence of non-consensual sex, were facilitators for positive SRHR outcomes.

Three studies [35–37] highlighted that a lack of social support networks within their neighbourhoods, having poor telephone networks, and poor road and physical infrastructure, collectively, was associated with delayed and reduced health-seeking behaviours, healthcare utilization, and were perceived by refugee women as a cause of poor mental health, including perinatal depression. Additionally, administrative and legal issues, alongside financial constraints that include incurring high out-of-pocket expenditure for accessing hospital services for refugee women living in urban settings, hindered healthcare access, increased health-seeking dropout rates, and poor mental health [35, 42]. One study reported that women relied on their husbands, who engaged in underpaid informal work, to afford their medical expenses. Contrary to this, having the UNHCR card and being connected to local NGOs reduced the fear of being arrested and/or detained and receiving healthcare and treatment without incurring out-of-pocket payments [42].

### Gendered Perspectives

Several studies also highlighted important gendered perspectives, which positively or negatively influenced healthcare access, health-seeking and utilization behaviours, and their health outcomes. Two studies [30, 33] reported that requiring spousal consent for health-seeking or husbands’ disapproval of contraceptives resulted in foregoing healthcare related to pregnancy care, STI checks, and contraceptive usage. Additionally, women’s financial dependence on their male family members and spouses influenced their healthcare decision-making regarding health-seeking and treatment [42].

The lack of their spouses’ involvement in child rearing contributed to women’s poor mental health as mothers often felt alone and overwhelmed. Strict gender norms such as veiling practices among Rohingya women were associated with the strong tendency for home deliveries and the reluctance to give birth at health facilities, especially given that health transport vehicles and facilities were primarily designed for mixed-sex use. Contrary to this, Barua et al. [32] highlighted that the recruitment of Rohingya and Bengali women as community health workers, compared to having male community health workers, increased health communication, reduced language barriers, and increased health-seeking behaviours related to pregnancy care.

## Discussion

This review aimed to scope the current evidence regarding the health and healthcare access of transit refugee women across South and Southeast Asia. The findings drew insights on the health outcomes, alongside the facilitators and barriers to healthcare access, health-seeking and healthcare utilization and knowledge of mainly the Rohingya communities residing in Bangladesh, Karen communities in Malaysia, and Chin communities residing Thailand. Given that South and Southeast Asia are regular migratory routes, our findings indicate that there is a dearth of evidence concerning transit refugees’ health and access to healthcare. Therefore, more evidence is needed to understand the needs, challenges, determinants, and experiences of transit refugee women to address and improve the health and health equity for these subgroup populations. Our findings also shed light on how gender perspectives influence the health and healthcare access of transit refugee women within these transit contexts. These perspectives provide important insights on both the need and ways to better integrate targeted gender-responsive strategies to improve the health and healthcare access for women refugees in transit.

The findings from this review revealed that transit women faced significant challenges, primarily related to their sexual and reproductive health and rights (SRHR), including contraceptive usage, family planning, STI and HIV prevention and control, as well as pregnancy and neonatal care. In addition, these women experienced poor psychological and mental health and were vulnerable to SGBV. SRHR emerged as a central theme in the review, as the majority of the studies focused on maternal and reproductive-aged women. In line with our findings, studies from other regions, including Sub-Saharan Africa, Europe and the Mediterranean have identified sexual and reproductive health as a central focus, with migration and displacement interrupting access to family planning, and sexual health and reproductive services and rights, placing women at greater risk of adverse health outcomes [43–45]. These findings therefore underscore the importance of prioritizing SHRH within the humanitarian crisis and refugee contexts, particularly in relation to women transit refugee populations.

The findings revealed how several health-determining factors either hindered or facilitated healthcare access, health-seeking and utilization behaviours, and health knowledge transmission. Corroborating wider studies, these findings highlighted the significant influence of physical distance from healthcare facilities, the availability of services, financial constraints, and out-of-pocket healthcare expenditure [13]. Additionally, having access to humanitarian assistance programs, such as the UNHCR card, which allows refugees to access healthcare services, medicines, and treatments, played a critical role in shaping healthcare access and health-seeking behaviours. However, these health facilitating and hindering determinants are closely linked to transit conditions, such as whether refugees reside in camps or in urban areas along with the host community. For example, in Cox’s Bazar, Bangladesh, Rohingya populations are placed in designated transit refugee camps that provide health clinics and services, including community health workers who address the health needs within the camp setting [29–33]. In contrast, other migrant populations residing in urban areas are required to seek their own healthcare, often resulting in high out-of-pocket expenses [42]. This financial burden impacts the regularity and frequency of healthcare utilization, particularly for sexual and reproductive health and rights (SRHR) needs, such as contraceptive use, family planning, STI control and HIV prevention, and maternal and neonatal care.

Religious beliefs, socio-cultural practices, and prescribed gender norms further compound the barriers to healthcare access and utilization [7]. For instance, among Rohingya communities, women’s veiling practices, prohibitions against opposite-sex interactions, stigmatization of reproductive health issues —including family planning and contraceptive use—and requirements for spousal consent for healthcare access are significant obstacles for transit women to seek treatment [30, 32, 33]. These barriers limit access to essential SRHR services and increase women’s susceptibility to sexual and gender-based violence and poor mental health outcomes.

### Study Limitations

We acknowledge two significant limitations in this study. First, due to the absence of a universal classification for identifying this transient subgroup, their refugee status is often poorly defined, especially in countries that serve as established transit hubs and offer provisions such as designated refugee camps. As a result, they may be categorized under refugee or asylum seeker status, which does not accurately reflect their situation as transit refugees. With this view, in some studies that included a mixed study population including non-refugees, a minimum of 25% threshold was applied to filter articles to ensure enough representation of refugees’ health outcomes [35–38]. Although these articles provide valuable insights into the healthcare access challenges faced by transit refugee women, the lack of clear definitions regarding their status may have limited our ability to draw stronger conclusions about transit women’s health inequities. Second, the search was limited to English-language articles. This restriction may have led to the exclusion of studies offering country-specific perspectives and experiences, potentially overlooking important nuances related to the gender perspectives that intersect with migration, transit refugee status, and health inequities.

As the population of transit refugees continues to grow globally, there is an urgent need for future research and policy action to address intersectional health challenges faced by transit refugee women, particularly across South and Southeast Asia. To address these challenges, health interventions targeting transit refugee women’s health issues must adopt context-specific and gender-responsive approaches that provide accessible, culturally sensitive, and comprehensive healthcare services [46]. Evaluations of health programs from Cox’s Bazar in Bangladesh that are designed to integrate more culturally appropriate and gender-responsive approaches, such as providing counselling and contraceptive consultations, along with the recruitment of women, including Rohingya women, as health volunteers and midwives, were found to significantly improves HIV control, increase contraceptive usage, improve family planning practices, ANC, safe abortion practices, and increase rates of facility-based deliveries compared to births occurring in shelters. Additionally, health programs that have engaged with spouses and male community members, including religious leaders, have improved women’s health-seeking, particularly in relation to their sexual and reproductive health, reduced incidence and risks of SGBV and child marriage outcomes [47].

## Conclusion

This scoping review highlights the significant health outcomes and issues related to healthcare access and health-seeking and utilization, faced by refugee women in transit across South and Southeast Asia, which are common migratory routes from countries of origin to host nations. Barriers such as distance and availability of healthcare services, high healthcare costs, stigmatization, alongside religious and gender-based restrictions, often result in delayed or foregone care, particularly for women’s sexual and reproductive health. These challenges are further compounded by poor mental health and heightened vulnerability to sexual and gender-based violence. Targeted interventions such as the provision of the UNHCR humanitarian cards, and gender-responsive approaches such as the recruitment of women healthcare workers, and the establishment of sex-specific health facilities, are shown to improve healthcare access and improve health outcomes. This review sheds light on the limited evidence available regarding the health and healthcare access of transit refugee women, with a regional focus of South and Southeast Asia. The review underscores the critical need for more gender-responsive and targeted interventions to improve access to essential healthcare for transit women refugees living in precarious conditions across the region.

## Data Availability

No datasets were generated or analysed during the current study.

## Appendix 1

See Table 2.

**Table 2:** List of key words used in the review

Key words [MeSH Terms]	Related words [MeSH Terms]
Transit refugee	refugees”[MeSH Terms] OR refugee [Text Word] · Refugee in transit, transit refugees (undocumented migrant, illegal immigrant, displaced persons, internally displaced person)
Healthcare access	Health Services Accessibility, Healthcare Access, Access to Care, Health Equity, Healthcare Disparities, Barriers to Healthcare, Health Service Availability, Geographic Accessibility, Financial Accessibility, Healthcare Affordability, Health Insurance Coverage, Primary Care Access, Rural Health Services, Urban Health Disparities · Health behaviours (including, health-seeking, health-preventative behaviour, health-promoting behaviour, healthcare utilization)
Health outcomes	Mental health (stress, depression, PTSD) Psychological wellbeing markers (e.g., suicidal ideation), Sexual and reproductive health and rights (including, family planning, contraceptive use, pregnancy, abortion, menopause menstrual health and hygiene) Food insecurity (or hunger, food scarcity) and Malnutrition (or Malnourishment, Undernutrition, Nutritional Deficiency, Nutritional Deficiencies) · Sexual and gender-based violence (domestic violence, intimate partner violence, violence against women)
Geography/countries	South Asian and Southeast Asian region (list of countries: Bangladesh, Bhutan, India, Maldives, Nepal, Pakistan, Sri Lanka, Afghanistan, Brunei, Cambodia, Indonesia, Laos, Malaysia, Myanmar, the Philippines, Singapore, Thailand, and Vietnam)

Example Search: (((transit refugee) AND (women)) AND (health)) AND (southeast asia):

(“transit”[All Fields] OR “transited”[All Fields] OR “transiting”[All Fields] OR “transition”[All Fields] OR “transitional”[All Fields] OR “transitionals”[All Fields] OR “transitioned”[All Fields] OR “transitioning”[All Fields] OR “transitions”[All Fields] OR “transits”[All Fields]) AND (“refugee s”[All Fields] OR “refugees”[MeSH Terms] OR “refugees”[All Fields] OR “refugee”[All Fields]) AND (“womans”[All Fields] OR “women”[MeSH Terms] OR “women”[All Fields] OR “woman”[All Fields] OR “women s”[All Fields] OR “womens”[All Fields]) AND (“health”[MeSH Terms] OR “health”[All Fields] OR “health s”[All Fields] OR “healthful”[All Fields] OR “healthfulness”[All Fields] OR “healths”[All Fields]) AND (“asia, southern”[MeSH Terms] OR (“asia”[All Fields] AND “southern”[All Fields]) OR “southern asia”[All Fields] OR (“south”[All Fields] AND “asia”[All Fields]) OR “south asia”[All Fields]).

Some keywords did not have corresponding MeSH terms. Therefore, these keywords were searched as free-text terms to ensure comprehensive coverage

## Appendix 2

See Table 3.

**Table 3:** Quality appraisal of studies included in the review

Checklist items	Study 1	Study 2	Study 3	Study 4	Study 5	Study 6	Study 7	Study 8	Study 9	Study 10	Study 11	Study 12	Study 13	Study 14
Clear inclusion criteria	✓	✓	✓	✓	✓	✓	✓	✓	✓	✓	✓	✓	✓	✓
Description of study subjects and setting	✓	✓	✓	✓	✓	✓	✓	✓	✓	✓	✓	✓	✓	✓
Valid and reliable exposure measurement	✓	✓	✓	✓	✓	✓	✓	✓	✓	✓	✓	✓	✓	✓
Use of measurement criteria	✓	✓	✓	✓	✓	✓	✓	✓	✓	✓	✓	✓	✓	✓
Identification of confounding factors	✓	✓	✓	✓	✓	Na	✓	Na	✓	✓	✓	✓	✓	✓
Stated strategies for confounding factors	✓	✓	✓	✓	✓	Na	✓	Na	✓	✓	✓	✓	✓	✓
Valid and reliable outcomes measurement	✓	✓	✓	✓	✓	✓	✓	✓	✓	✓	✓	✓	✓	✓
Use of proper statistical analysis	✓	✓	✓	✓ Mixed-methods analysis	✓	Qualitative analysis	✓	Qualitative analysis	✓	✓	✓	✓	✓	✓
Overall risk rating and inclusion	Low risk & included	Low risk & included	Low risk & included	Low risk & included	Low risk & included	Low risk & included	Low risk & included	Low risk & included	Low risk & included	Low risk & included	Low risk & included	Low risk & included	Low risk & included	Low risk & included

## Appendix 3

See Table 4.

**Table 4:** Summary of articles included in the review

Author (Year)	Country (Region)	Sample population	Study design	Area of health focus	Key outcomes	Notes (e.g., intersectional perspectives)
Khan et al. [30]	Bangladesh (South Asia)	N = 508 Rohingya refugee women	Cross-sectional study	Health knowledge about HIV	Knowledge about HIV transmission increased with age at which they were first married, with having formal education, with engaging in work outside their households, and among those whose husbands were employed Knowledge about HIV transmission was associated and increased with the availability of healthcare services where women resided HIV transmission and prevention influenced by (reduced) mobility, cultural and religious restrictions/customs regarding access to reproductive healthcare	
Khan et al. [30]	Bangladesh (South Asia)	N = 493 (Rohingya refugee women, included women who were married or given birth 2 years prior to the survey)	Cross-sectional study	Family planning and access to contraceptives	Over half of the sample were using contraceptives at the time of the study, with the largest proportion reportedly using contraceptive injection, followed by oral contraceptive pill, an implant, and undergone sterilization Reasons for not using contraceptives: disapproval by women’s husbands, desiring pregnancy, and religious beliefs/restrictions Only 10.95% of the sampled women received frequent and recommended home visits (every 2 weeks) by a healthcare worker, and those who received these visits received both family planning and provision of contraceptives Use of contraceptives were higher among those who reported no non-consensual sex with their husband in the year before, compared with occasional or frequent non-consensual sex, among those who resided in a camp with a mobile healthcare centre, compared with women whose camp did not have health facilities Women’s employment outside their household and the existence of a healthcare centre within the camp was positively associated with the use of contraceptives Women whose recent pregnancy was unplanned and lower age at first marriage was negatively associated with the use of contraceptives	
Zakaria et al. [31]	Bangladesh (South Asia)	N = 415 (Rohingya women from Cox’s Bazar refugee camp, Bangladesh, included women were of reproductive aged (18–49 years)	Cross-sectional study	Knowledge about SRH related to: STIs, family planning, menstrual health and hygiene, maternal health	SRH awareness was associated with: The current number children (having fewer children were associated with better SRH outcomes) Having a reliable source of SRH knowledge Having access to SRH and MH care Ever had consulted a doctor/nurse on SRH Ever had a door-visit by a NGO worker on SRH Participated in a SRH awareness program Barriers to SRH awareness and healthcare: Language barrier between doctors working in the refugee camps Religious prohibitions and beliefs Cultural and traditional practices/beliefs (e.g., not cooking or engaging in domestic chores during menstruation)	The recruitment of Rohingya and Bengali women as community health workers increased health communication and reduced language barriers Working with Imam (Muslim religious leader) and Majhi (local Rohingya leaders) helped to disseminate health information and messages, including at Mosque and religious institutions
Barua et al. [32]	Bangladesh (South Asia)	1st phase (N = 100 Rohingya refugee women who used services) 2nd phase (n = 33, of which 12 were Rohingya refugee women) who used services	Mixed-methods study	Maternal health, including emergency obstetric services	Enablers to healthcare access: Knowledge about the presence of referral hubs, having counselling services, and having transportation to the hubs improved maternal health indicators Sources of knowledge including community health volunteers, followed by neighbours, and community leaders (Majhi) improved maternal health indicators Barriers to healthcare: Mis or poor knowledge about the transport option reduced health-seeking and utilization behaviours Cultural and gender norms—women are not allowed to speak with male health workers Poor telephone network reduced health-seeking and utilization behaviours Poor roads and infrastructure delayed timely healthcare and increased physical and psychological distress among women	Due to strict gender norms and veiling practices among Rohingya women, there is a strong tendency for home deliveries and reluctance to give birth at health facilities (due to mixed-sex health vehicles and spaces within the facilities) Male community health workers cannot interact with Rohingya women due to cultural and gender norms, and Rohingya women tend not to use the transportation vehicles during pregnancy related care due to male drivers
Azad et al. [33]	Bangladesh (South Asia)	N = 400 Rohingya women (18–49 years) who had lived with their husbands and delivered at least one child at least 1 year before the study	Cross-sectional study	Family planning and contraceptive usage	Reasons for not using family planning and contraceptives: Husband’s disapproval Desire to get pregnant Religious/cultural belief that using contraceptives was committing a sin Being worried about probable side effects of contraceptives Belief that more children brought financial solvency to the family Belief that bearing children until a male child is born, agreeing that having more sons would ensure security for elderly parents Perception that contraceptive usage reduced pleasure of sexual intercourse Factors contributing to family planning and contraceptive use Women being employed (having a profession), and having less children Having a physician/nurse as a source of family planning knowledge Having and participating in family planning interventions at the camp Regular visitations to the clinic/health facility and discussing SRH issues with a healthcare provider	
Rajaratnam and Aznam [42]	Malaysia (Southeast Asia)	N = 22 Rohingya women, N = 11 key informants (medical social workers, medical officers, volunteer workers, refugee officers and a mental health provider)	Qualitative study	Sexual Gender-based violence, mental health	Factors leading to healthcare access: Having a UNHCR card helped to reduce the fear of being arrested and detained Having a UNHCR card helped to receive treatment, and avoid high out-of-pocket payments for treatment and healthcare Having a connection with any NGOs or local Malaysian individuals to pay their medical bills lead to better access to hospital and healthcare Barriers to healthcare access: Administrative issues (e.g., long waiting queues for UNHCR card registration) posed significant challenges in relation to admission and treatment in (public) hospitals High out-of-pocket expenditure for hospital treatment (e.g., surgical procedures), childbirth, and buying medicines Stigma and threats from health professionals in relation to payment of medical expenses High payment charges for consultations and medicines, and regular visitations increased drop-out rates and mental illness	Common form of SGBV were marital rape, domestic violence, child rape and sexual assaults (perpetrators included their husbands, community members, locals, employers and teachers) Women and girls engaged in unpaid domestic work and depended on their male family members and husbands for money to afford medical expenses Social support from other women and family/friends relieved psychological distress and mental health outcomes
Perera et al. [34]	Thailand-Myanmar border	N = 34,240 women who had a delivery between 2008 to 2019	Retrospective study (2008–2019)	Family planning and maternal health	Low birth weight deliveries were associated with fewer first and second trimester ANC visits, compared with normal birth weight deliveries The number of first and second trimester ANC visits and prior utilization of family planning services were protective factors against low birthweight deliveries Mothers who delivered low birthweight infants were likely to have lower utilization rate of family planning services Low birthweight deliveries were significantly associated with mothers age likely to be under 20 years of age	
Fellmeth et al. [35]	Thailand-Myanmar border	N = 116 (29 pregnant refugees, 63 pregnant migrants, 24 antenatal clinic staff)	Qualitative study	Perinatal mental health	Symptoms and behaviours related to mental illness among women included, intense sadness, anger and fear, excessive worry, and physical symptoms (i.e., headaches, loss of appetite, poor or excessive sleep, heart palpitations, and having cold hands and feet Causes of perinatal mental illness included lack of employment and low income, lack of family support (i.e., unsupportive partners, parents, and family members) and worry about children Suicide was not perceived as not being attributed to mental illness. The causes of suicide included: shame, guilt, economic and family issues, spirituality	
Fellmeth et al. [36]	Thailand-Myanmar border	N = 568 women (317 labour migrants and 250 refugees)	Mixed method cohort study	Perinatal mental health	Psychosocial factors such as interpersonal violence, a history of trauma, a self-reported history of depression, perceived insufficiency of social support, and labour migrant status were the main risk factors for moderate/severe perinatal depression Depression of any severity, including moderate/severe depression was higher among refugees compared to labour migrants At one month postpartum, women who experienced any depression during the first trimester are more likely to still be depressed, while those who had moderate-severe depression have generally begun to recover Antenatal and postnatal depression were significantly associated, emphasizing the importance of early identification and intervention to avoid persistence of depression	
Fellmeth et al. [37]	Thailand-Myanmar border	N = 451; 233 (52%) pregnant migrants and 218 (48%) pregnant refugees;	Mixed method prospective cohort study	Perinatal mental health	The prevalence of perinatal depression was higher among refugees at 47.3%, compared to 38.6% among migrants Proportion of refugees who reported support from partners and sufficient support were lower than that of migrants, while refugees also exhibited lower received support scores than migrants Insufficient perceived support among refugees increased the odds of perinatal depression (AOR: 1.89) History of depression (AOR 3.35) and high trauma score (AOR 1.63) increased the risk of depression, while having a telephone halved the odds of depression	
Fellmeth et al. [37]	Thailand-Myanmar border	N = 568; 318 (56%) migrant and 250 (44%) refugees in refugee and migrants’ clinics	Mixed method prospective cohort study	Perinatal mental health	Prevalence of suicidal ideation during perinatal period was 5.3%, while subthreshold symptoms was identified in 23.8% participants Determinants of suicidal ideation includes household size, plan to return to Myanmar, interpersonal violence, history of depression, and social support Trauma score (aOR 2.32) and unplanned pregnancy (aOR 2.74) increased the odds of suicidal ideation Women with suicidal ideation were 24 times more likely than women without suicidal ideation to have a diagnosis of depression Refugees were more likely to experience suicidal ideation than migrants	
Prins et al. [40]	Thailand-Myanmar border	N = 522	Cross sectional study	Maternal and child health	Knowledge: Most participants could recognize jaundice (92.5%) and knew that it was harmful (95%). Most also reported they would bring the baby to the clinic if jaundice was observed (96.2%). Most commonly cited source of knowledge was healthcare workers (65.9%) Practice: 41.4% of the pregnant women reported the use of mothballs, though only 18.2% used them to store baby clothes and blankets. Furthermore, 46.7% used menthol-containing products on baby skin Parous women who previously had a child with neonatal jaundice were more likely to identify the cause of jaundice, but did not exhibit safer behaviour such as reducing the use of mothballs and menthol which acts as haemolytic agent that increases the risk of jaundice in populations with a high prevalence of glucose-6-phosphate dehydrogenase deficiency,	
Falb et al. [39]	Thailand	N = 377 women (reportedly had a partner/married and had most recent pregnancy that ended in a live birth within the last 2 years of the study)	Cross-sectional study	Gender-based violence and maternal health	Symptoms of pregnancy complications such as feeling very weak of tired, vaginal bleeding, severe abdominal pain, or blurred vision was found in: 50% of women who experienced both conflict victimization and intimate partner violence (IPV) victimization One in three women who reported conflict victimization only one in five women who reported IPV victimization only One in three women who reported one type of victimization event The odds of experiencing pregnancy complication symptoms increased with reports of: Any type of violence (aOR 3.1) Conflict victimization, (aOR 3.0) History of abortion or stillbirth (aOR 1.6)	Health seeking behaviour was observed in 90% of women who reported symptoms of pregnancy complications in their last pregnancy that ended in a live birth
Srikanok et al. [41]	Thailand	N = 35.139 new family planning consultations	Retrospective study (1996–2015)	Family planning and contraceptive usage	In both TBBC and UNHCR population estimates, the number of new contraceptive consultations per 1000 reproductive age women from 2002 to 2015 increased The type of contraceptive most widely used were depo-injections, followed by pills which were trending up, and condoms which were trending down The use of IUD and implant increased by up to 8 × and 4 × respectively, along with upward trends in female sterilization uptake that surpassed LARC after 2004 Pregnancy rate is showing a downward trend, with its lowest peak at 137 pregnancies per 1000 reproductive age women from 2009 to 2015 Miscarriage rates remained relatively stable, with an apparent decrease in recent years	

## Abbreviations

ANC: Antenatal care
FGM: Female genital mutilation
HIV: Human immunodeficiency virus
IOM: International organization for migration
SGBV: Sexual and gender-based violence
SRHR: Sexual and reproductive health and rights
STIs: Sexually transmitted infections
UHC: Universal health coverage
UNHCR: United Nations high commissioner for refugees
WHO: World health organization
